# Preparation and Testing of Polyethylenimine-Impregnated Silica Gel for CO_2_ Capture

**DOI:** 10.3390/gels10060360

**Published:** 2024-05-23

**Authors:** Veronika Kyselová, Jakub Havlín, Karel Ciahotný

**Affiliations:** 1 Department of Sustainable Fuels and Green Chemistry, University of Chemistry and Technology Prague, Technická 5, 166 28 Prague, Czech Republic; karel.ciahotny@vscht.cz; 2 Central Laboratories, University of Chemistry and Technology Prague, Technická 5, 166 28 Prague, Czech Republic; jakub.havlin@vscht.cz

**Keywords:** silica gel, polyethylenimine, carbon dioxide capture, adsorption, desorption

## Abstract

This work studied the low-temperature sorption of carbon dioxide on impregnated silica gel. An impregnating agent was used polyethyleneimine. The content of the impregnating agent in the silica gel matrix was 33.4 wt.%. Material properties such as the Brunauer–Emmett–Teller (BET) surface area, pore distribution, total pore volume, and thermal stability of the impregnated material were determined for the sample. During the measurement of the adsorption–desorption cycles, the loss of the impregnating agent in the material matrix was also determined. Due to the decrease in the content of polyethyleneimine, the sorption capacity of the adsorbent for CO_2_ also decreased. It was found that after the 20th adsorption–desorption cycle, the content of the impregnating agent in the adsorbent dropped by 3.15 wt.%, and, as a result, the adsorption capacity for CO_2_ dropped to almost half.

## 1. Introduction

Carbon dioxide from anthropogenic sources is considered one of the main greenhouse gases responsible for global warming. More than 65% of anthropogenic CO_2_ comes from fossil fuel combustion. To limit the Earth’s surface temperature rise to less than 2 °C, it is imperative to prevent the increase in greenhouse gas emissions, as agreed in the United Nations Climate Change Conference Paris 2015. Therefore, it is necessary to develop new technologies for clean energy production with minimal CO_2_ emissions. One of the ways to reduce CO_2_ emissions is CCS (carbon capture and storage) technologies. These technologies are based on carbon dioxide capture, transportation, and storage [1,2]. Particular emphasis is placed on carbon dioxide capture. Currently, a series of carbon dioxide capture technologies are available. Large-scale implementation of these technologies is limited due to technological, economic, environmental, and safety issues. Some of the technologies applied for the absorption of CO_2_ on an industrial scale use washing liquids containing organic amines as an active agent. However, this process is energetically demanding, and the aqueous amine solution is corrosive to construction materials. Therefore, other technologies are being developed for carbon dioxide capture for CCS processes. These technologies are based on the use of adsorption, membrane separation, and cryogenic separation [2].

Chemical absorption in an aqueous amine solution or adsorption in suitable sorbent materials is among the most advanced and widespread post-combustion technologies for carbon dioxide capture. Carbon dioxide removal via absorption in an aqueous amine solution is a widely used technology. Capture in the aqueous amine solution takes place because of the high affinity of the sorbent for CO_2_. In this process, a chemical bond is formed between the working amine solution and CO_2_ [3,4,5].

The saturated amine solution can be thermally regenerated. During the regeneration process, the formed chemical bond breaks, and the captured CO_2_ is released. The thermal regeneration process demands high energy consumption to heat the washing solution to a high temperature and break the bond between the absorption agent and the bounded CO_2_ [6,7]. The most commonly used solvents are monoethanolamine (MEA), diethanolamine (DEA), and methyl diethanolamine (MDEA). Other solvents that can also be used are piperazine or ammonia [8,9,10,11]. The regeneration of the saturated washing solution is carried out with steam at temperatures in the range of 100–120 °C. Steam condenses in the stripping column, and high-purity CO_2_ leaves the column [7]. This process was originally used to remove CO_2_ from natural gas. However, the compositions of natural gas and flue gas are very different. The use of amine solutions for CO_2_ removal from flue gas presents a series of problems, e.g., high energy consumption for the regeneration of the working solution, degradation and losses, as well as negative impacts on the environment [12,13].

The removal of carbon dioxide from flue gases via adsorption technologies possesses great potential. For the separation of carbon dioxide from flue gases, it is necessary to select a suitable adsorbent that has a high adsorption capacity and selectivity for CO_2_ and that can be easily regenerated. Carbonaceous adsorption materials have outstanding properties, and due to their hydrophobic nature, show very good selectivity in a humid flue gas environment [14,15]. A very important role during the adsorption process is played by the interaction of the adsorbed CO_2_ and the sorbent [16]. Carbon dioxide-saturated adsorption materials can be regenerated by increasing the temperature using the temperature swing adsorption (TSA) process or reducing the pressure using the pressure swing adsorption (PSA) process. If the treated flue gas has a low carbon dioxide content, the TSA process can be used. However, the main disadvantage of the TSA process is the relatively time-consuming desorption cycle due to the need for sorbent cooling.

Carbon dioxide adsorption on solid sorbents is a less energetically demanding process than capture in amine solutions. Suitable materials for CO_2_ capture are continuously being tested and developed [17].

When using conventional adsorbents, the physical adsorption of CO_2_ is based on van der Waals forces. Therefore, their adsorption capacity for CO_2_ significantly decreases with an increasing adsorption temperature and decreasing CO_2_ concentration in the treated gas [18]. When these adsorbents are used to remove CO_2_ from flue gases at temperatures above 40 °C, the adsorption capacities for CO_2_ are already low, requiring the construction of large adsorbers and frequent regeneration of the adsorbent. Therefore, the process becomes economically disadvantageous. Therefore, modified adsorbents are being developed that bind CO_2_ to their inner surface based on chemical bonding. Organic amines used in absorption technologies serve as modifying agents. In Li Wenhao and Fu Dong’s study, they dealt with the capture of carbon dioxide from flue gas by solid amine adsorbents [19].

For amine impregnation, commonly available porous materials with a large surface area and a large pore volume can be used, for example, porous silica, zeolites, activated carbon, metal–organic frameworks (MOFs), and other materials such as bentonite and activated γ-alumina [20,21,22,23,24].

When activated carbon is used as the matrix, the porous carbon with the amine has a relatively low retention, resulting in poorer selectivity for the carbon dioxide under low-temperature sorption in the temperature range of 50–120 °C [3]. In contrast, amine-impregnated zeolites are highly hydrophilic materials, and thus, their sorption capacity is reduced in a high-humidity environment. MOFs have a high sorption capacity for pure CO_2_ without impurities and at high pressures [25].

Impregnated silica gel sorbents are considered very suitable for the capture of CO_2_ from flue gas due to their high adsorption capacity. Furthermore, impregnated silica gel possesses not only a good CO_2_ adsorption capacity but also good resistance to the presence of contaminants. One way to improve the properties of the sorbent is the incorporation of more nitrogen functional groups into its porous structure. This can be achieved by silica gel impregnation with polymer amines such as polyethylenimine (PEI) [26,27,28].

This work is part of long-term research focused on the development of functionalized adsorbents, which is being carried out at our department. Several modified adsorbents with different polyethylenimine concentrations were prepared and tested [29,30]. This study was also carried out by Lin Li et al., who dealt with the preparation of impregnated silica gel with different concentrations of polyethyleneimine in the matrix [31].

In this study, silica gel impregnated with a high content of polyethyleneimine was prepared as a CO_2_ adsorbent. The porous structural properties of the modified adsorbent were determined using measurements of the internal surface area, adsorption pore volume, and pore size distribution. The content of polyethyleneimine in the adsorbent was determined by elemental analysis. The thermal stability of the polyethyleneimine in the adsorbent was determined by thermogravimetric analysis. The modified adsorbent was tested in repeated cycles for CO_2_ adsorption and the subsequent regeneration of the saturated adsorbent.

## 2. Materials and Methods

Silica gel impregnated with a high content of polyethylenimine was prepared under laboratory conditions as a sorbent for the capture of CO_2_. The impregnated sorbent was sampled and used for elemental analysis and the measurement of the specific surface area, pore volume, and pore distribution. Furthermore, the sorbent adsorption capacity for CO_2_ was measured at different temperatures using a Quantachrome ASiQ analyzer. The sample was then tested at the selected temperature of 50 °C for 20 adsorption–desorption cycles. The sample used was subsequently subjected to all individual analyses to determine changes caused by the effect of long-term exposure to CO_2_ exposure on the sorbent properties and composition of the sorbent.

### 2.1. Preparation of the Sample

The very porous silica gel labeled SGR 50 (Silcarbon Aktivkohle, Kirchhundem, Germany) was selected as the basic sorption material to undergo impregnation. For the impregnation process, polyethylenimine (PEI), Mn = 600 g·mol^−1^, and methanol (Sigma Aldrich, St. Louis, MO, USA) were used.

Before impregnation, fresh silica gel sorbent was activated in the air for 12 h at 200 °C and atmospheric pressure. Activation was necessary to degas and dehydrate the sorbent. Several impregnation methods have been described in a series of published works. The selected impregnation procedure was the one that bonded the most polyethylenimine to the sorbent [16,28,32].

The solution containing 45% polyethylenimine in methanol was prepared and mixed for 30 min. Subsequently, the activated silica gel was infused into the solution and left there for 5 h. Subsequently, the mixture was evacuated for 10 min and then aerated for 5 min. The described process of evacuation and aeration was repeated three times. The excess solution was filtered, and the impregnated material was left to dry for 12 h at 70 °C under atmospheric pressure [33].

### 2.2. Analysis

#### 2.2.1. BET Specific Surface Area and Pore Distribution

The specific surface area was measured using the Coulter SA 3100 analyzer (Beckman Coulter, CA, USA). The analyzer works based on the physical adsorption of N_2_ from the gaseous phase in the sorbent at a temperature of 77 K. Each sample was weighed and inserted into a sample tube. Subsequently, the samples were outgassed for 240 min at 110 °C under high vacuum. After outgassing, the sample tubes containing the samples were weighed again and placed into the analyzer’s measuring site to undergo evacuation again. Once evacuation was over, the sample tubes were immersed in liquid nitrogen and dosed with precisely calculated volumes of gaseous nitrogen. As soon as the adsorption equilibrium was stabilized, the equilibrium pressure was measured in order to calculate the quantity of adsorbed nitrogen. Applying this procedure, the adsorption isotherm of nitrogen was measured at a relative pressure of 0 to 0.99 and a temperature of 77 K, and then the desorption isotherm was measured under the same conditions. The pressure decreases in the sample tubes containing the samples was achieved by gradually aspirating known gaseous nitrogen volumes. Measurements were evaluated based on the shapes of the adsorption and desorption isotherms. For the range corresponding to a relative pressure of 0–0.3, the quantity of adsorbed nitrogen was evaluated based on the Brunauer–Emmett–Teller (BET) equation. From the obtained coefficients, the BET specific surface areas of each sample were determined. The total pore volumes were determined by the quantity of adsorbed nitrogen at a relative pressure close to one. The pore size distributions were calculated from the desorption isotherms based on the Barrett–Joyner–Halenda (BJH) equation using the Kelvin model.

#### 2.2.2. Elemental Analysis

In order to determine the amount of polyethylenimine in the impregnated sorbents, elemental analysis was performed using a Flash 1112 analyzer (Thermo Fisher Scientific, Waltham, MA, USA). This analyzer determines the percentage of carbon, hydrogen, nitrogen, sulfur, and, eventually, oxygen (CHNS-O) in the analyzed sample. Initially, the analyzed sample undergoes combustion in a sample crucible under oxygen flow at high temperatures. The generated sample combustion products are then catalytically reduced, purified, separated on a short chromatographic column, and quantified using a thermal conductivity detector (TCD).

#### 2.2.3. Measurement of CO_2_ Adsorption Using Quantachrome AsiQ

The carbon dioxide adsorption on the sorbent at different temperatures was measured using a Quantachrome ASiQ (Boynton Beach, FL, USA) analyzer. The selected temperatures were 30, 50, 80, and 100 °C. The samples weighed approximately 0.5 g. The activation of each sample was carried out by placing the measuring cell with the sample in a stream of nitrogen at 110 °C for 4 h.

The sample tube was then evacuated, cooled at room temperature, and weighed to find the weight loss after activation. Subsequently, sorbent testing at the selected temperature in a pure carbon dioxide atmosphere took place. Precise carbon dioxide volumes were gradually dosed into the sample tube. Once equilibrium was reached, the actual quantity of adsorbed CO_2_ could be determined. Subsequently, known quantities of CO_2_ were dosed into the sample tube to measure the next points of the adsorption isotherms. The sample tube containing the sample was then cooled to room temperature and weighed to find the weight gain. The last step was the regeneration of the sorbent performed under the same conditions as activation, i.e., for 4 h in a nitrogen stream at 110 °C. Based on the data evaluation of the tests performed at different temperatures, one of the temperature values was chosen to perform multiple CO_2_ adsorption–desorption cycle testing. Sorbent testing cycles using pure carbon dioxide and consecutive regeneration were repeated until a constant process efficiency was reached.

The equipment recorded the total gas volume of carbon dioxide at the required temperature and pressures in the range of 100–800 Torr. This volume represented the total volume used for both chemisorption and physisorption. After adsorption, the saturated material was evacuated for 120 min to remove the physically trapped amount from the sample (the evacuation of the sample was sufficient to break the van der Waals bonds and release the carbon dioxide), and the equipment measured the amount of released CO_2_ gas. Molecules of CO_2_ captured by chemisorption were firmly attached to the surface of the material, and to break the chemical bonds, more energy was needed in the form of an increase in temperature to 110 °C. The amount of CO_2_ released by heating the sample was also recorded by the equipment.

#### 2.2.4. TGA

Thermogravimetry (TGA) combined with evolved gas analysis (EGA) was used to follow the thermal stability and decomposition of the impregnant in the silica gel structure. TG analysis was performed using a Pyris (Perkin Elmer, Waltham, MA, USA) coupled with FTIR (Perkin Elmer, USA). The experiment was carried out in the temperature range of 40 °C to 215 °C in a nitrogen atmosphere with a flow rate of 60 mL·min^−1^. The heating rate was 10 K·min^−1^.

#### 2.2.5. SEM Analysis

Microscopic analysis of the adsorbent samples was performed using an SEM TESCAN Lyra3 GMU (Brno, Czech Republic) scanning microscope equipped with an auto-emission cathode (FEG) in the secondary electron imaging mode.

## 3. Results and Discussion

The sorbent adsorption properties and adsorption capacity of carbon dioxide were improved by modifying the inner surface of the material. Sorbent modification was achieved by loading the impregnant into the porous system and bonding it to the inner surface of the adsorption material. Polyethylenimine, suitable for the chemical sorption of carbon dioxide, was used as an impregnant.

**Figure 1:**
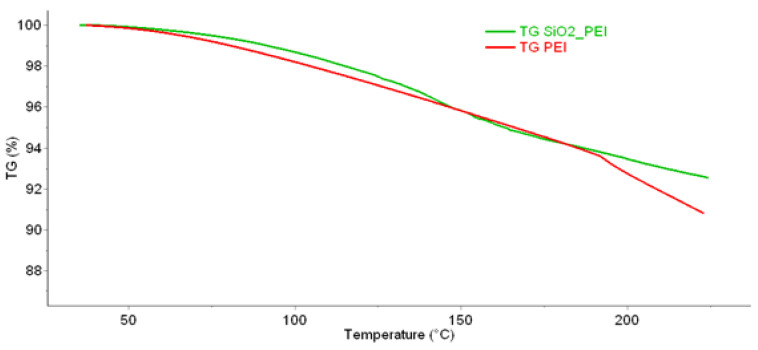
TGA analysis of the prepared impregnated material and pure PEI, measured on the Pyris 1 TGA in the temperature range of 40 to 215 °C.

The sample was subsequently subjected to several analyses to determine the stability of the material and the impregnating agent in the silica gel matrix. One of the ways to determine temperature stability is thermogravimetric analysis (TGA). The following Figure 1 shows a comparison of TGA for pure PEI and PEI on silica gel.

**Figure 2:**
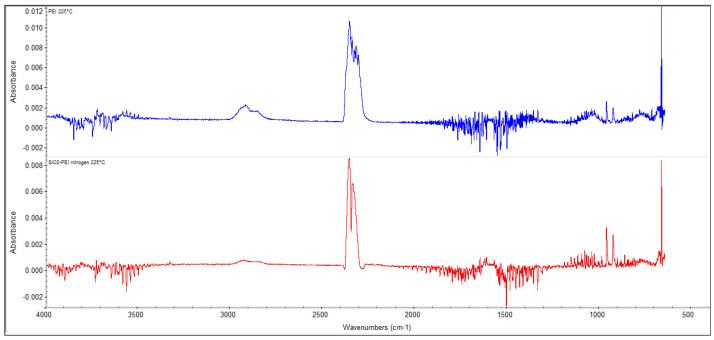
TG-FTIR analysis results of pure polyethylenimine and impregnated material.

Figure 2 shows the spectrum characterizing the composition of the released products when pure PEI was heated, which was recorded at a temperature of 220 °C. The peaks at the wavelengths of 966 and 932 cm^−1^ are specific to ammonia, and the peaks at the wavelengths of 2928, 1044, and 771 cm^−1^ are specific to ethylenediamine. Figure 2 shows the same spectrum measured when heating a PEI-impregnated silica gel sample. From the comparison of the two spectra shown in Figure 2, it is clear that the products of material decomposition originated in the PEI in both cases.

**Figure 3:**
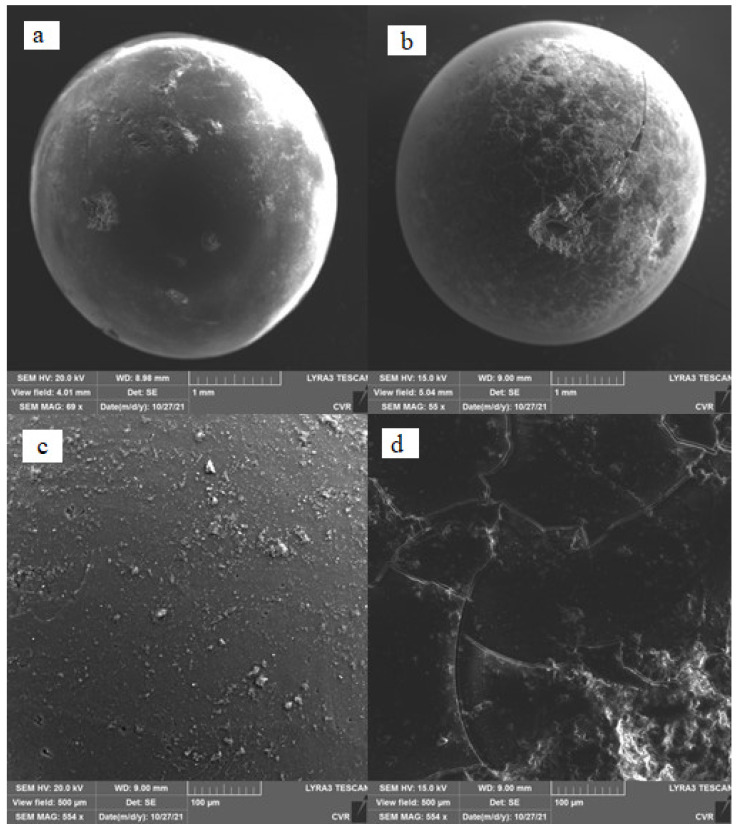
Comparison of samples using SEM. (**a**) and (**c**): pure SiO_2_; (**b**) and (**d**): impregnated SiO_2_.

Figure 3 shows a comparison of SEM photographs of pure SiO_2_ (a) and impregnated SiO_2_ (b). Significant changes are seen on the surface of the entire sample particle; the surface layer is cracked. This phenomenon is clearly visible in the enlarged photograph (d). It can be seen in the photos that polyethyleneimine was probably applied mainly on the outer surfaces of the silica gel particles. Because it had a different thermal expansion from silica gel, the structure of the polyethylenimine cracked when the impregnated adsorbent sample was heated.

Other analyses included measurements of the BET surface area, pore volume, and pore distribution, as well as elemental analysis, which served to determine the content of the impregnation agent in the material matrix. The results are summarized in the following tables.

In Table 1, a comparison of the BET specific surface areas and total pore volumes of the non-impregnated and impregnated silica gels are depicted. It can be seen that the BET specific surface area and total pore volume significantly decreased after sorbent impregnation, which confirmed the presence of polyethylenimine in the porous system.

**Figure 4:**
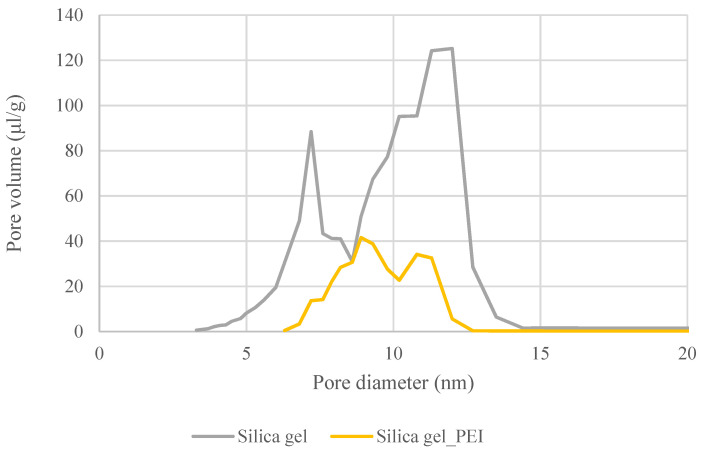
Comparison of pore size distributions in pure silica gel and PEI-impregnated SiO_2_.

The following Figure 4 shows the changes in the distributions of pores in the non-impregnated and impregnated silica gels. The changes were caused by the settling of the impregnating agent in the silica gel matrix.

The polyethylenimine content in the sample was determined using elemental analysis (by the increases in the nitrogen and hydrogen contents in the sample matrix). The results obtained from the elemental analysis are shown in Table 1.

The first adsorption tests were performed with a Quantachrome ASiQ instrument in an atmosphere of pure carbon dioxide using non-impregnated silica gel. The results of the pure carbon dioxide adsorption measurements are shown in Table 2.

The significant decrease in the adsorption capacity of silica gel for CO_2_ with an increasing temperature confirms that the majority of CO_2_ was captured on the non-impregnated silica gel by the mechanism of physical adsorption.

The test results using the PEI-modified adsorbent and the Quantachrome ASiQ analyzer are presented in Table 3. The adsorption capacity for CO_2_ was 10.26 g·100 g^−1^ sorbent at a temperature of 30 °C. By comparing Table 2 and Table 3, it can be seen that the incorporation of polyethyleneimine into the matrix of the material resulted in a reduction in the BET surface area of the impregnated material. On the contrary, the BET surface area of the pure silica gel was measured to be almost 400 m^2^·g^−1^.

The values of the BET specific surface areas and the total pore volumes are higher than for the same sample before the start of the tests (see Table 3). This indicates that further cracking of the outer layer of PEI occurred during the testing, thereby exposing some additional pores inside the adsorbent particles.

**Figure 5:**
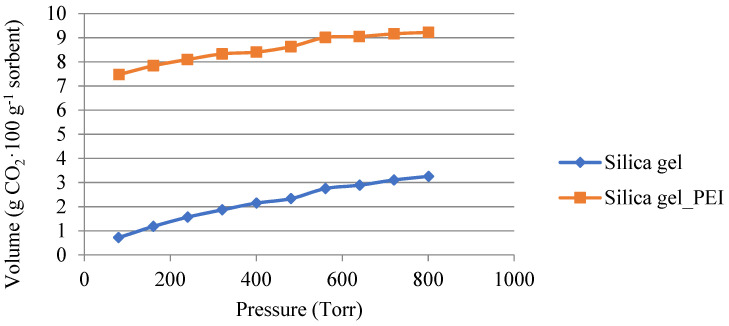
Adsorption isotherms of carbon dioxide for non-impregnated and impregnated SiO_2_ at 50 °C.

The comparison of the adsorption isotherms measured with pure carbon dioxide at a temperature of 50 °C for the non-impregnated SiO_2_ and impregnated SiO_2_ is shown in Figure 5.

**Figure 6:**
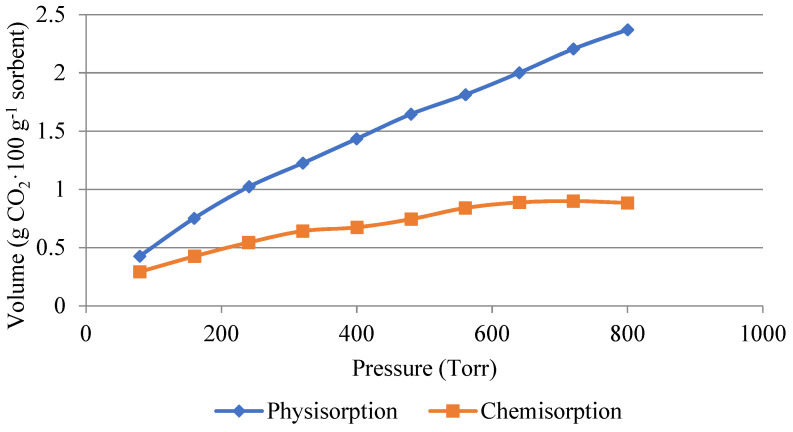
Volume of carbon dioxide used for physisorption and chemisorption in the non-impregnated sample.

**Figure 7:**
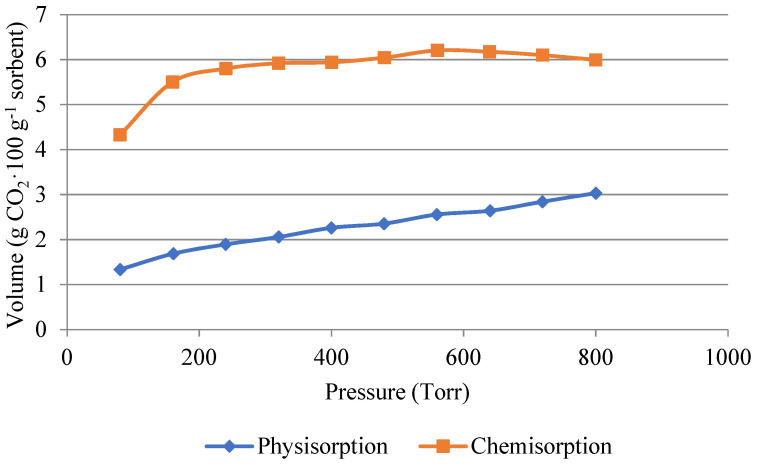
Volume of carbon dioxide used for physisorption and chemisorption in the impregnated sample.

The following Figure 6 and Figure 7 show a comparison of the amount of carbon dioxide sorbed by physisorption and chemisorption. The measurements were carried out with pure carbon dioxide at a temperature of 50 °C. Figure 6 shows the amount adsorbed on the non-impregnated silica gel. It can be seen that physisorption dominated during the process. The physically absorbed amount was around 2.5 g·100 g^−1^ of adsorbent. The amount of carbon dioxide in the 1 g·100 g^−1^ of sorbent was sorbed by chemisorption, i.e., it was a reversible process at higher temperatures.

The opposite phenomenon is shown in Figure 7, where in the case of the impregnated material, carbon dioxide was mainly trapped in the silica gel matrix by chemisorption. The amount of 6 g·100 g^−1^ of carbon dioxide was captured by chemisorption. Therefore, for desorption, it is necessary to heat the material to a temperature above 100 °C for the carbon dioxide to desorb.

According to the results depicted in Table 3, the maximum adsorption capacity was reached at 30 °C. In real operating conditions, it would be possible to cool the flue gas and reach a flue gas temperature of 50 °C. Additionally, the adsorption capacity at 50 °C decreased by 1 g·100 g^−1^ sorbent compared with the adsorption capacity at 30 °C. Therefore, multiple CO_2_ adsorption–desorption cycle tests were performed with the sorbent SiO_2__PEI at the selected temperature of 50 °C. It can be seen in Table 4 that increasing the temperature resulted in a further decrease in the adsorption capacity. This behavior is in agreement with experimental results published in another work that reported a considerable decrease in the adsorption capacity from 65 °C [34].

**Figure 8:**
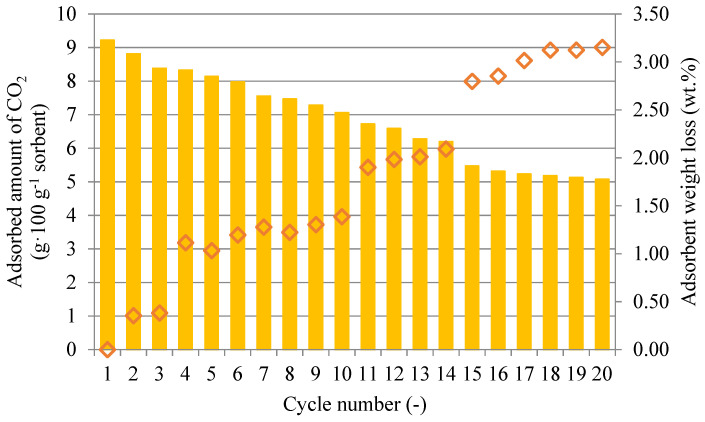
Sorbent SiO_2__PEI CO_2_ adsorption capacity and sorbent weight loss for each cycle at 50 °C.

Figure 8 illustrates the changes in the CO_2_ sorption capacity at 50 °C by repeated adsorption–desorption cycles for the sorbent SiO_2__PEI. Once the sorbent was saturated, the desorption of captured CO_2_ in the nitrogen stream at 110 °C was initiated. Figure 8 shows that after the 15th cycle, no substantial changes in the sorbent adsorption capacity were observed. After 20 cycles, the sorbent adsorption capacity fell by 45% due to impregnant loss during desorption at 110 °C, which was confirmed by the decrease in the impregnant content in the sample matrix.

Figure 8 also shows the weight loss of the sorption material during the course of each adsorption–desorption cycle. The gradual total sorbent weight loss was most probably caused by impregnant loss from the matrix, resulting in a decreased CO_2_ adsorption capacity. After the 20th cycle, the sorbent weight loss was 3.15%. This result was confirmed by the elemental analysis of the sample shown in Table 4.

The increase in the BET surface area of the impregnated material after 20 cycles was due to the evaporation of the impregnating agent from the material. Therefore, there was an increase from the value of 98 to 150 m^2^·g^−1^.

**Figure 9:**
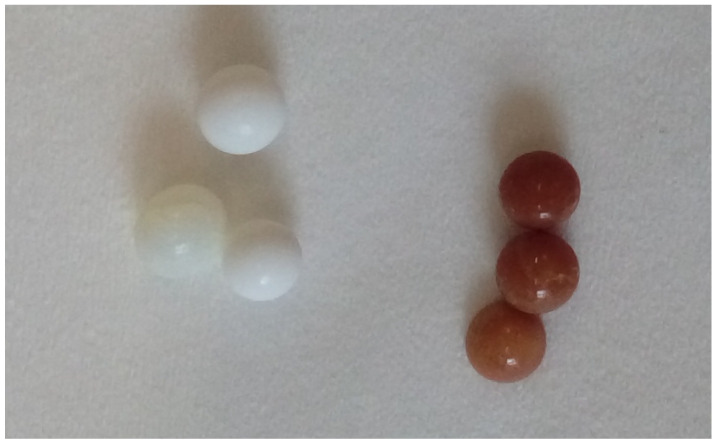
Sorbent SiO_2__PEI before (**left**) and after 20 cycles at 50 °C (**right**).

Figure 9 also shows the visual changes that occurred in the sample after 20 cycles at 50 °C (compared with the original impregnated material). The color change was caused by heating the impregnated material to a temperature above 100 °C.

## 4. Conclusions

The highly porous silica gel, commonly used as a CO_2_ sorption material, modified by polyethylenimine, was tested for low-temperature CO_2_ sorption. The quantity of polyethylenimine present in the porous system was estimated by means of an elemental analysis of the impregnated sample. The amount of polyethyleneimine in the silica gel matrix was determined to be 34.3% by weighing.

Material properties such as the BET specific surface area, total pore volume, and pore distribution were determined for the non-impregnated and impregnated sorbent. The results acquired confirmed the presence of the necessary amount of the impregnant in the porous structure of the sorbent. Increasing the polyethylenimine content resulted in a reduction in the BET specific surface area and a decrease in the total pore volume. There was a decrease in the surface area from 416 to 98 m^2^·g^−1^ and a decrease in the total pore volume. In the case of the impregnated material, the pore volume was reduced by a third in comparison with that of the pure non-impregnated material.

During tests of the CO_2_ adsorption capacities at temperatures of 30 to 100 °C, a significant drop in the adsorption capacity was already observed for the non-impregnated silica gel at a temperature of 50 °C. During the measurement with pure carbon dioxide at a temperature of 30 °C, a sorption capacity of 7.44 g·100 g^−1^ of the adsorbent was achieved. On the contrary, at a temperature of 50 °C, a significantly lower sorption capacity of 3.26 g·100 g^−1^ of the adsorbent was achieved. This significant decrease was not measured in the impregnated sample, where the sorption capacity decreased by 1.23 g·100 g^−1^ of the adsorbent at the same temperatures. The sorption capacity at a temperature of 50 °C was 9.03 g·100 g^−1^ of the adsorbent. Therefore, subsequent adsorption–desorption cycles tests were carried out with the impregnated material at a temperature of 50 °C, which is a temperature that could be used in industrial practice to capture carbon dioxide from flue gases.

An important finding in this work is the comparison of the chemisorption and physisorption of carbon dioxide on impregnated and non-impregnated silica gel. It was proven here that low-temperature sorption of carbon dioxide onto impregnated silica gel takes place mainly by chemisorption (6 g·100 g^−1^ of the adsorbent was bound by chemisorption). Therefore, higher temperatures are required for CO_2_ desorption. In this work, the desorption temperature of 110 °C was chosen; at this temperature, according to TGA, there is no high loss of the impregnating agent in the impregnated material. It is, therefore, obvious that the impregnation of this material is suitable for industrial use as it increases the adsorption capacity for low-temperature adsorption of carbon dioxide at a temperature of 50 °C or higher.

## Figures and Tables

**Table 1:** Comparison of elemental analysis results of SiO_2_ and SiO_2__PEI samples.

Sample	N Content (wt.%)	C Content (wt.%)	H Content (wt.%)	PEI Content (wt.%)
Pure SiO_2_	0	0.22	0.72	0
SiO_2__PEI	11.07	18.64	4.62	34.33

**Table 2:** Sorbent SiO_2_: maximal pure CO_2_ sorption capacity at different temperatures.

Temperature (°C)	Adsorption Capacity (g/100 g Sorbent)	BET Specific Surface Area (m^2^·g^−1^)	Total Pore Volume (mL·g^−1^)
30	7.44	396	0.988
50	3.26	396	0.984
80	2.80	396	0.997
100	0.68	397	0.979

**Table 3:** Sorbent SiO_2__PEI CO_2_ sorption test results at different temperatures.

Temperature (°C)	Adsorption Capacity (g/100 g Sorbent)	BET Specific Surface Area (m^2^·g^−1^)	Total Pore Volume (mL·g^−1^)
30	10.26	114	0.389
50	9.03	115	0.315
80	8.37	120	0.314
100	8.13	100	0.348

**Table 4:** Elemental analysis of sorbent SiO_2__PEI before and after 20 adsorption–desorption cycles.

Sample	N Content (wt.%)	C Content (wt.%)	H Content (wt.%)	BET Specific Surface Area (m^2^·g^−1^)	Total Pore Volume (mL·g^−1^)
SiO_2__PEI before testing	11.07	18.64	4.62	98	0.3030
SiO_2__PEI after 20 cycles	8.59	16.26	3.20	151.44	0.2995

## Data Availability

The data presented in this study are available upon request from the corresponding author.
